# Antiviral Activity of Germacrone against Pseudorabies Virus in Vitro

**DOI:** 10.3390/pathogens8040258

**Published:** 2019-11-22

**Authors:** Wanting He, Xiaofeng Zhai, Jingyin Su, Rui Ye, Yuna Zheng, Shuo Su

**Affiliations:** Joint International Research Laboratory of Animal Health and Food Safety, Engineering Laboratory of Animal Immunity of Jiangsu Province, College of Veterinary Medicine, Nanjing Agricultural University, Nanjing 210000, China; 2018107065@njau.edu.cn (W.H.); 2019207027@njau.edu.cn (X.Z.); 2018107064@njau.edu.cn (J.S.); 2018807153@njau.edu.cn (R.Y.); emerald.aimo@gmail.com (Y.Z.)

**Keywords:** PRV, germacrone, antiviral

## Abstract

Pseudorabies virus (PRV), a member of the *Herpesviridae*, is the causative agent of an acute infectious disease in a variety of animals. The emergence of a novel variant strain brought huge economic losses to the pig industry since classical vaccine strains were not completely effective against variant strains. Therefore, the development of new anti-pseudorabies virus drugs and vaccines is of great significance for the treatment and prevention of pseudorabies. In this study, we found that germacrone, one of the major components of the essential oils extracted from Rhizoma Curcuma, was able to effectively inhibit PRV replication in a dose-dependent manner in vitro. Germacrone showed antiviral activity against PRV in the early phase of the viral replication cycle. Moreover, we found that germacrone does not directly kill the virus, nor does it affect the expression of the PRV receptor protein nectin-1, nectin-2, and CD155. Our results suggest germacrone could be used as an efficient microbicide or immunomodulatory agent in the control of the emerging variant PRV.

## 1. Introduction

Pseudorabies (PR), an acute, febrile infectious disease caused by pseudorabies virus (PRV), usually occurs in sows and is characterized by reproductive disorders and neurological signs [1]. PRV was discovered at the beginning of the 20th century and is prevalent in at least 44 countries [2]. Pigs are the natural hosts and the main source of transmission, but PRV has a broad host range and can also infect a variety of livestock and wildlife [2,3]. Recently, it has been reported that PRV can infect humans, with weakness, fever, sweating, dysphagia, and neurological dysfunction as typical symptoms [4]. PRV belongs to the *Alpherpesvirus* subfamily [5,6]. PR has been eradicated in several countries including the USA, New Zealand, and many European countries. However, PRV is still in circulation worldwide.

Highly pathogenic PRV variants emerged in several pig farms in China in late 2011 [7,8]. In previous reports, we analyzed all known PRV full-length genomes and all available gB, gC, gD, and gE gene sequences. We found that PRV could be divided into clade 1 and 2 based on the gC gene. The current vaccine strain Bartha-K61 clustered with clade 1. However, the most recent PRV variants clustered with clade 2. We previously reported that variant PRV forms and recombination in China could be the source of continued epidemics [9,10]. So far, the most effective method of prevention and control of pseudorabies is vaccination. The emergence of PRV variant strains brought huge economic losses to the pig industry since classical vaccines were not completely effective against it [11,12]. The fact that PRV can persist in the host for a long time after infection makes its prevention and control challenging [13].

The development of treatment with effective and safe antiviral drugs has become imperative since PRV vaccines do not provide complete protection. Due to the large specific surface area and unique physical and chemical properties of nanomaterials, some researchers studied the effect of nanomaterials on antiviral activity. In particular, graphene oxide significantly reduced PRV replication at noncytotoxic concentrations [14]. In addition, it has been reported that lithium chloride (LiCl) and diammonium glycyrrhizinate (DG) have good inhibitory effect on PRV replication [15]. Of note, although there are some studies on the antiviral effect of LiCl and DG on PRV, relatively few studies on their antiviral effect against newly emerged PRV variant virus were reported. Considering the currently wide prevalence of PRV variant in China, effective antiviral drugs against the variant PRV should be screened as soon as possible.

Germacrone is a monocyclic sesquiterpene extracted from gingeraceae plants with antitumor, anticancer, antiviral, antibacterial, and anti-inflammatory activity [16,17,18]. In particular, germacrone antitumor effect on the human hepatoma cell line is mediated via G2/M cell cycle arrest and by promoting apoptosis [19]. In terms of antiviral activity, germacrone inhibits influenza virus (IAV), Porcine parvovirus (PPV), Feline calicivirus (FCV), and Porcine reproductive and respiratory syndrome virus (PRRSV) replication [16,20,21,22]. Germacrone has inhibitory effects on H1N1, H3N2 influenza viruses, and Influenza B viruses in the early stages of the viral cycle and can protect mice from fatal infection [20]. However, it does not block PRRSV adsorption and invasion and cannot inhibit both classical and highly pathogenic II PRRSV strains [16].

The objective of this study was to explore the antiviral effects of germacrone on PRV infection and its antiviral mechanism. We found that germacrone can inhibit PRV replication in a dose-dependent manner at the early stage of viral replication.

## 2. Materials and Methods

### 2.1. Cells and Virus

Vero and LLC-PK-1 cells were cultured in Dulbecco’s Modified Eagle Medium (DMEM) supplemented with 10% fetal bovine serum (FBS, Cellsera Australia) and cultured at 37 °C in a CO_2_ humidified incubator. Variant PRV and PRV vaccine strain Barth K61 were propagated in Vero cells cultured in DMEM supplemented with 2% FBS. The viral titers were titrated in Vero cells.

### 2.2. Chemicals, Antibodies and Other Reagents

Germacrone (CAS 6902-91-6) was purchased from Weikeqi (China) and dissolved in dimethyl sulfoxide (DMSO) at 20 mM. Lithium chloride (LiCl) (Sigma, St. Louis, MO, USA) and diammonium glycyrrhizinate (DG) (TargetMol, USA) were diluted in DMEM at 1 M and 10 mg/mL concentration, respectively, and filter-sterilized. The following primary antibodies were purchased from commercial resources: rabbit anti-β-Actin pAb from HuanBio (China); mouse anti-PRV-gB mAb (LD-DW-Z0022-2) from lv du (China). The secondary antibodies used in western blotting, including DyLight 800 goat anti-mouse IgG (H+L) (074-1806) and DyLight 800 goat anti-rabbit IgG (H+L) (074-1506), were purchased from KPL (Gaithersburg, MD). Secondary antibody used in confocal microscopy, including goat anti-mouse–FITC (Fluorescein isothiocyanate) (172-1806) was obtained from KPL. The lactate dehydrogenase (LDH) cytotoxicity assay kit (C0017) was purchased from Beyotime (China).

### 2.3. Cytotoxicity Assay

Vero or PK-1 cells were seeded in 96-well plates. After the cell confluence reached 90%, cells were incubated with different concentrations of germacrone (0, 10, 50, 100, 150, 200, 250 μM) or DMSO at 37 °C for 36 h. At the end of the incubation period, cell cytotoxicity was determined using the LDH cytotoxicity assay kit, according to the manufacturer’s instructions. The optical density (OD) value of each well at a wavelength of 490 nm was determined using an automatic microplate reader (TECAN Infinite^®^ 200 PRO).

### 2.4. Virus Infection and Viral Titer Assay

Variant PRV and PRV vaccine strain Barth K61 were propagated in Vero cells. To infect, Vero cells were incubated with PRV for 1h, then washed with serum-free DMEM and incubated in DMEM supplemented with 2% FBS for 48 h. Virus titration or subsequent infectivity analysis was titrated in Vero cells. Briefly, Vero cells were seeded in 96-well plates. After the cell confluence reached 90%, the culture medium was discarded and the cells were washed with serum-free DMEM. The virus was 10-fold serially diluted. After 1 h, the virus was discarded and the unbound virus particles were washed away with serum-free DMEM. Culture medium containing 2% FBS was added, and cells were cultured at 37 °C until obvious cytopathic effect (CPE) developed. After a further 48 h of incubation, the 50% tissue culture-infected dose (TCID_50_) was calculated by the Reed–Muench method.

### 2.5. DNA and RNA Extraction and Real-Time PCR

The TIANamp Genomic DNA Kit (TIAN GEN DP304) was used for DNA extraction. Total RNA from Vero or LLC-PK1 cells was extracted using TRIzol (Vazyme), and 1 μg cDNA was synthesized using the Thermo Scientific Revert Aid First Strand cDNA Synthesis Kit (Thermo Scientific) according to the manufacturer’s instructions. The SYBR Green master mix (Vazyme) was used to quantify the abundance of the target mRNA according to the manufacturer’s instructions. The procedure for real-time PCR consisted of an initial step at 95 °C for 5 min, followed by 40 cycles of 95 °C for 10 s, 60 °C for 30 s, and 95 °C for 15 s. Specific primers are shown in Table 1. The PRV viral gB gene level was determined by absolute quantification real-time PCR. Nectin-1, nectin-2, and CD155, which are believed to be the PRV receptors, were quantified by quantitative PCR. The relative level of RNA expression was determined by the 2^−ΔΔCT^ method. β-actin mRNA level was used as a loading control. The mean RNA level of the negative control (NC) group was set at 100%.

### 2.6. Indirect Immunofluorescence Assay (IFA)

Vero cells were seeded in 96-well plates. After the cell confluence reached 90%, the culture medium was discarded and the cells were washed with serum-free DMEM. Vero cells were infected with PRV at MOI 0.1 for 1 h, and the unbound virus particles were washed with serum-free DMEM. After removing unbound virus, 150 μM germacrone was added for 24 h. The cells were washed with phosphate-buffered saline (PBS), fixed with 4% paraformaldehyde, and permeabilized with 0.2% Triton X-100 for 30 min. The fixed cells were then incubated with primary antibodies (mouse anti-PRV-gB mAb, 1:1000) for 2 h, washed five times with PBS, and incubated with secondary antibodies (goat anti-mouse–ITC) for 1 h at 37 °C in the dark. After washing five times with PBS, the cells were incubated with DAPI (4′,6-diamidino-2-phenylindole) for 15 min to stain the nuclei. Images were acquired by using a fluorescence microscope (Nikon) with a video documentation system.

### 2.7. Western Blotting

Western blot analysis was performed as described previously [23]. Briefly, the cells were washed three times with serum-free DMEM and lysed in NP-40 lysis buffer. After concentration, equal amounts of protein (20 ug) were separated by 15% sodium dodecyl sulfate polyacrylamide gel electrophoresis (SDS-PAGE) and transferred to the nitrocellulose (NC) membranes. The NC membrane was blocked with 5% skim milk in PBS added 0.1% Tween 20 (PBST) for 1 h at room temperature and incubated with specified primary antibodies overnight. After being washed with PBST for five times, the membrane was incubated with secondary antibodies (DyLight 800 goat anti-mouse IgG or DyLight 800 goat anti-rabbit IgG) and subjected to LI-COOdyssey infrared image scanner.

### 2.8. Statistical Analysis

All the experiments were performed in triplicate, and the results are presented as means ± standard deviation (SD). The significance of differences between the drug- and negative control (NC)-treated groups was determined using the GraphPad Prism 7.0 software by a one-way ANOVA or unpaired t-test. Ns, no significant difference (* *p* < 0.05; ** *p* < 0.01; *** *p* < 0.001). The normal distributions of the data were confirmed by the Shapiro–Wilk normality test.

## 3. Results

**Figure 1:**
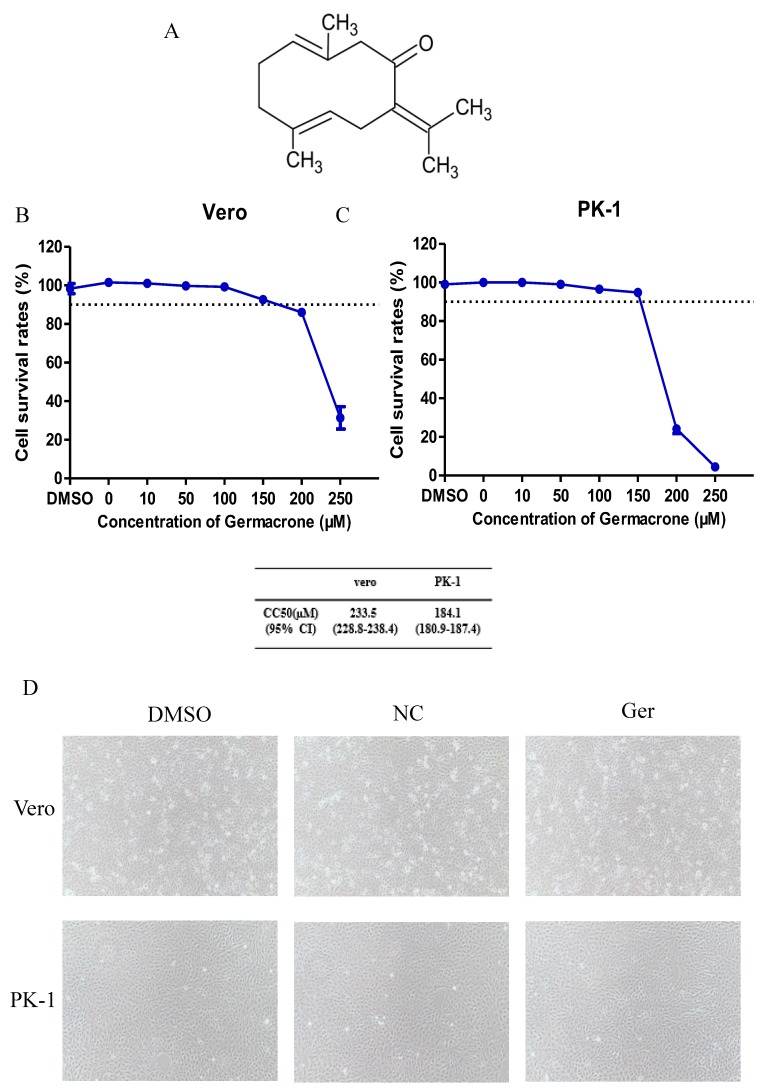
Nontoxic concentrations of germacrone on Vero and PK-1 cells. (**A**) Chemical structure of germacrone. (**B**,**C**) Percentage cell viability after treatment with different concentrations of germacrone (0, 10, 50, 100, 150, 200, 250 μM) in Vero (**B**) and PK-1 cells (**C**). The cell survival rates under different concentrations of drugs are given, and 90% above cell survival rate (over dotted line) is regarded as a nontoxic concentration of drugs. (**D**) Cell morphology of Vero and PK-1 cells in the negative control (NC)-, DMSO-, and germacrone-treated groups. The data are expressed as the mean ± SD of three independent experiments.

Germacrone is one of the main constituents of volatile oil from *rhizoma curcuma* [24]. The structure of germacrone is shown in Figure 1A. The concentration of germacrone that reduced cell viability by 50% was considered the 50% cytotoxic concentration (CC_50_). The cytotoxic effect of germacrone on Vero and PK-1 cells was determined using the LDH cytotoxicity assay kit. For both cell lines, the CC_50_ values were 233.5 and 184.1 µM germacrone in Vero and PK-1 cells, respectively (Figure 1B,C). The relative cell viability was above 90% after treatment with 150 μM germacrone. No changes in cell morphology were detected at 150 μM germacrone (Figure 1D).

**Figure 2:**
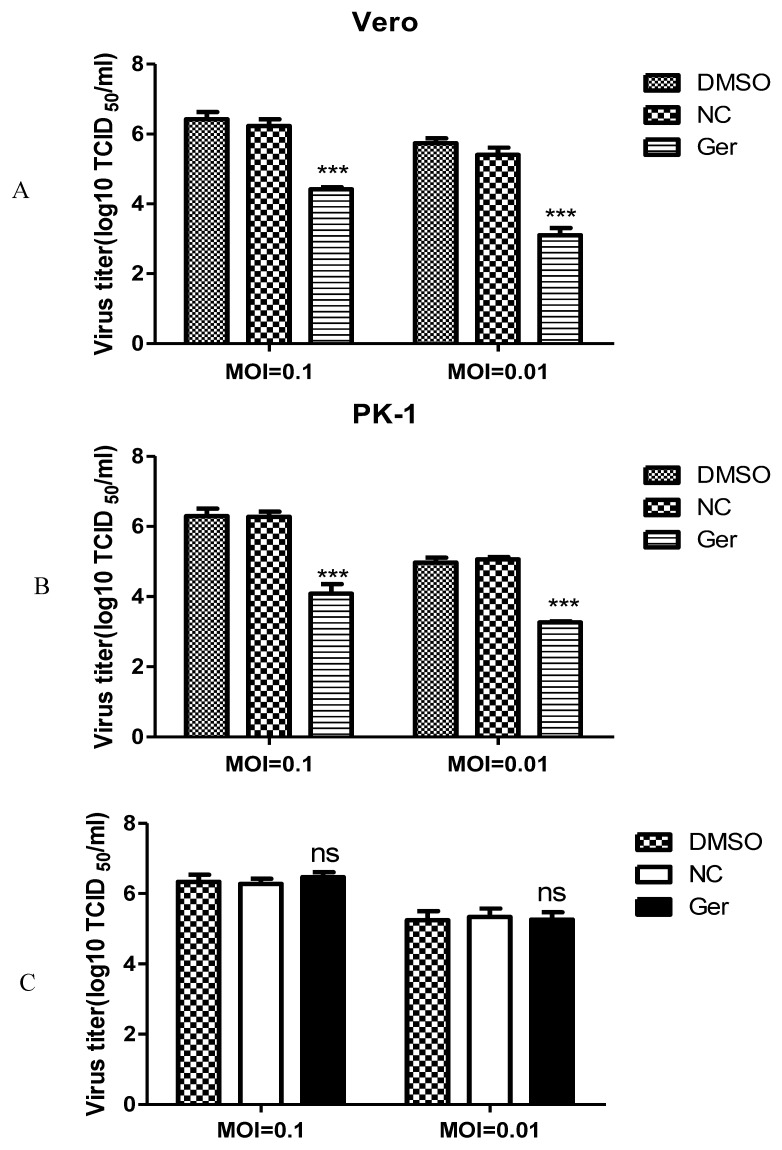
Germacrone reduces the production of infectious PRV particles and is not viricidal. (**A**,**B**) Vero (**A**) and PK-1 (**B**) cells were infected with pseudorabies virus (PRV) (MOI 0.1) for 1 h. The unbound virus particles were washed with serum-free DMEM, then medium containing 150 μM of germacrone was added. DMSO was used as a control. After 24 h of treatment, virus yielded in supernatants from infected cells was determined by TCID_50_ (50% tissue culture-infected dose) in Vero cells. (**C**) PRV was incubated with 150 μM of germacrone or DMSO at room temperature for 1 h. After incubation, the infectious titer was determined by TCID_50_ in Vero cells. Data are presented as the mean ± SD from triplicate samples. All the results were confirmed by three independent experiments (*** *p* < 0.001).

To evaluate whether germacrone could inhibit PRV replication, Vero or PK-1 cells were infected with PRV at a MOI of 0.01 or 0.1 for 1 h and treated with 150 μM germacrone for 24 h. Germacrone significantly inhibited viral replication compared with control cells and DMSO treatment (Figure 2A,B). In order to identify if germacrone has a direct negative effect on virions, we incubated virions with 150 μM of germacrone for 2 h at room temperature and determined the infectious titer by TCID_50_ on Vero cells. Germacrone did not influence PRV infectivity at a MOI of 0.01 or 0.1, indicating that germacrone is not viricidal (Figure 2C).

**Figure 3:**
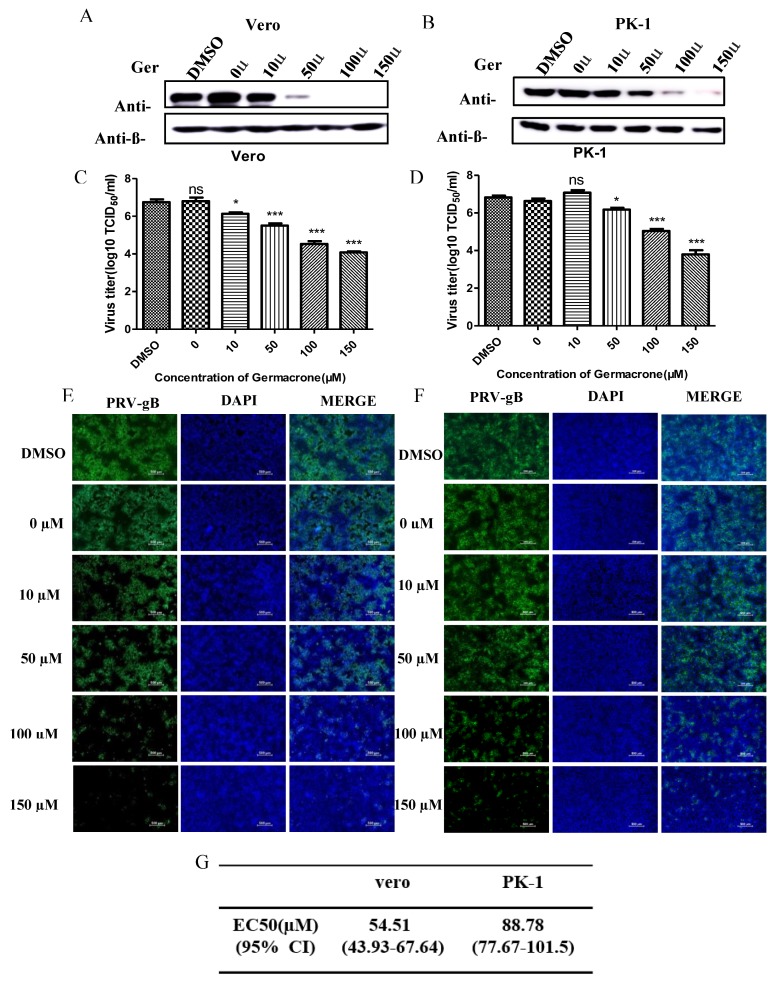
Germacrone can inhibit PRV replication in a dose-dependent manner. (**A**,**B**) Cells were infected with PRV (MOI 0.1) for 1 h. The unbound virus particles were washed with serum-free DMEM, then medium containing different concentrations of germacrone (0, 10, 50, 100, and 150 μM) was added. DMSO was used as a control. After 24 h of treatment, the level of gB protein was detected by western blot in Vero cells (**A**) or PK-1 cells (**B**). (**C**,**D**) The virus yielded in supernatants from infected cells was quantified by TCID_50_ in Vero cells. (**E**,**F**) The effect of different concentrations of germacrone on PRV, shown by IFA at 24 hpi in Vero cells (**E**) or PK-1 cells (**F**). Scale bar, 250 μm. (**G**) EC_50_ (50% effective concentration) values were calculated with GraphPad Prism software. Data are presented as the mean ± SD from triplicate samples. All the results were confirmed by three independent experiments (* *p* < 0.05, *** *p* < 0.001).

Next, to obtain detailed insight into the efficacy of germacrone against PRV, we tested the effect of germacrone in a dose-dependent manner on Vero and PK-1 cells. Treatment with 10–150 μM of germacrone inhibited PRV-gB protein levels and viral proliferation in Vero cells (Figure 3A,B). On the other hand, treatment with 10 μM of germacrone did not affect PRV-gB protein levels nor virus yield in PK-1 cells; however, treatment with 50–150 μM germacrone inhibited PRV-gB protein levels and viral proliferation in PK-1 cells (Figure 3C,D). In addition, a strong virus-positive signal was observed in negative control- and DMSO-treated cells 24 hpi in Vero and PK-1 cells by IFA. However, the fluorescent signal decreased after treatment with germacrone in a dose-dependent manner (Figure 3E,F). The obtained titer values were used for estimation of the 50% effective concentration (EC_50_). To obtain detailed insight into the efficacy of germacrone against PRV, we next calculated the EC_50_ values, which reflect the concentration of germacrone that is required to abolish infectious virus particle production by 50%. Figure 3G shows the EC_50_ values for germacrone following infection with PRV in Vero or PK-1 cells. The EC_50_ values were 54.51 and 88.78 µM germacrone in Vero and PK-1 cells, respectively. These results indicate that germacrone treatment resulted in a significant reduction in both virus titer and PRV-gB protein levels in a concentration-dependent manner.

**Figure 4:**
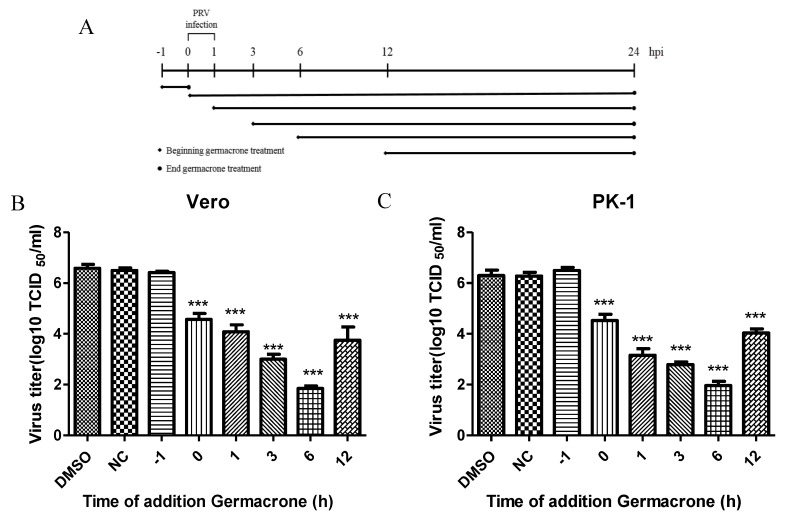
Germacrone inhibits PRV replication at an early stage. (**A**) Outline of the experiment. (**B**) Vero cells were infected with PRV (MOI 0.1), followed by treatment with 150 μM of germacrone at the indicated time (hpi). DMSO was used as a control. Virus titers were determined at 24 hpi. Data are presented as the mean ± SD from triplicate samples. All the results were confirmed by three independent experiments (*** *p* < 0.001).

To further illustrate the effect of germacrone on PRV infection, we performed time-of-addition assays (Figure 4A). Germacrone was added to Vero or PK-1 cells pre-, during or post-infection. In pretreatment experiments, cells were incubated with germacrone for 1 h, after which the compound was washed out, and then cells infected with PRV. In the “during” condition, germacrone and virus were added together to cells and 1 hpi the medium was removed, cells were washed, and continued to incubate with DMEM containing 150 μM germacrone. In the post-treatment experiments, cells were infected with PRV for 1 h, and after removing the PRV inoculum, cells were washed and germacrone was added at 1, 3, 6, and 12 hpi. In pre-, during or post-infection experiments, cells were infected at a MOI of 0.1 and germacrone was added at a final concentration of 150 μM. DMSO-treated cells were used as control. We observed that germacrone reduced PRV infectivity in the “during” or “post-infection” experiments (Figure 4B,C). No antiviral effect was observed when cells were pre-incubated with germacrone. This indicates that the antiviral effect of germacrone occurs at the early stage of PRV replication.

**Figure 5:**
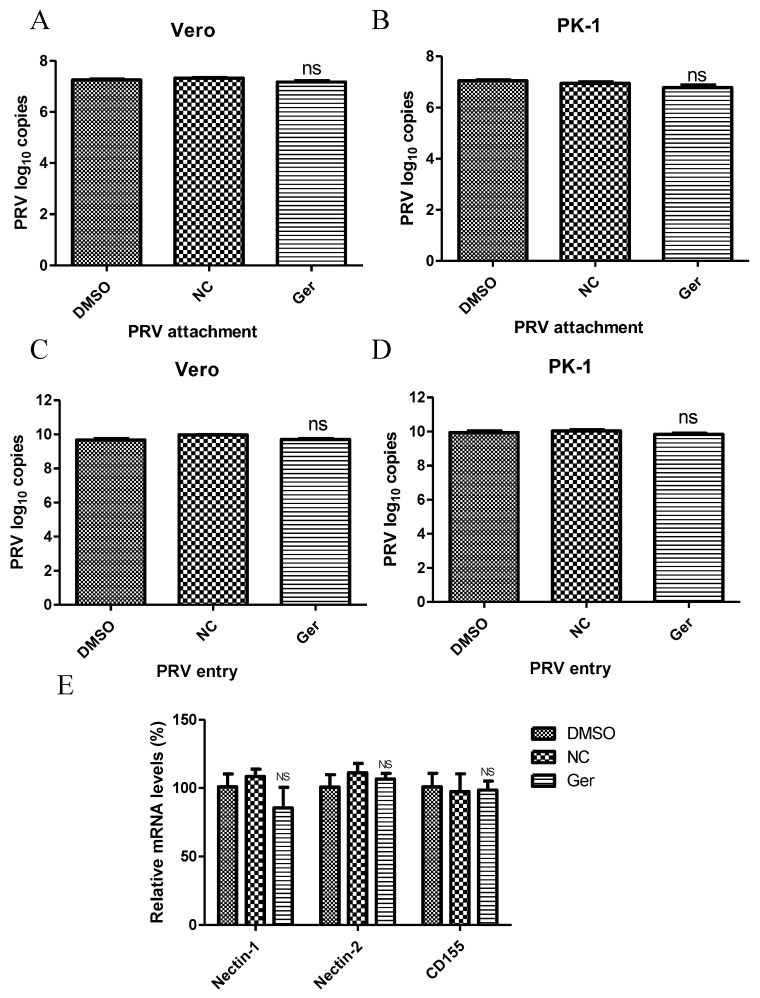
Germacrone treatment does not affect PRV attachment or entry. (**A**,**B**) Vero cells were exposed to the complex of virus particles (MOI 10) and germacrone or DMSO for 1 h at 4 °C at the attachment stage. After the unbound virus particles were washed with serum-free DMEM, the viral genome load was quantified by Q-PCR by amplification of the PRV gB gene. (**C**,**D**) Vero cells were inoculated with PRV at MOI 10 for 1 h at 4 °C, and then germacrone (150 μM) was added for 1 h at 37 °C. After washing three times with PBS (pH = 2.5), the viral genome load was quantified by Q-PCR by amplification of the PRV gB gene at 6 hpi. Values are represented as the mean ± SD of three independent experiments. Ns represents no significant difference. (**E**) The effect of germacrone on the nectin-1, nectin-2, and CD155 was measured by Q-PCR.

Next, we investigated the effect of germacrone on virus attachment and entry. Germacrone (150 μM) and PRV (MOI 10) were added together to cells and incubated for 1 h at 4 °C. The viral genome load was then quantified by Q-PCR via amplification of the PRV gB gene. We found no difference in drug-treated and negative control-treated cells (Figure 5A,B), suggesting that germacrone does not affect viral attachment to cells. Next, cells were inoculated with PRV at a MOI of 10 for 1 h at 4 °C, and then germacrone (150 μM) was added for 1 h at 37 °C to allow virus entry. The cells were then washed three times with PBS (pH = 2.5) to remove bound virus [25]. After 6 hpi, the viral genome load was quantified by Q-PCR via amplification of the PRV gB gene. No difference was found between germacrone- or DMSO-treated cells and negative control-treated cells (Figure 5C,D), suggesting that the germacrone does not affect virus entry. We also tested the effect of germacrone on nectin-1, nectin-2, and CD155, which are believed to be the PRV receptors, by quantitative PCR. Germacrone had no influence on nectin-1, nectin-2, and CD155 gene levels [26,27] (Figure 5E).

**Figure 6:**
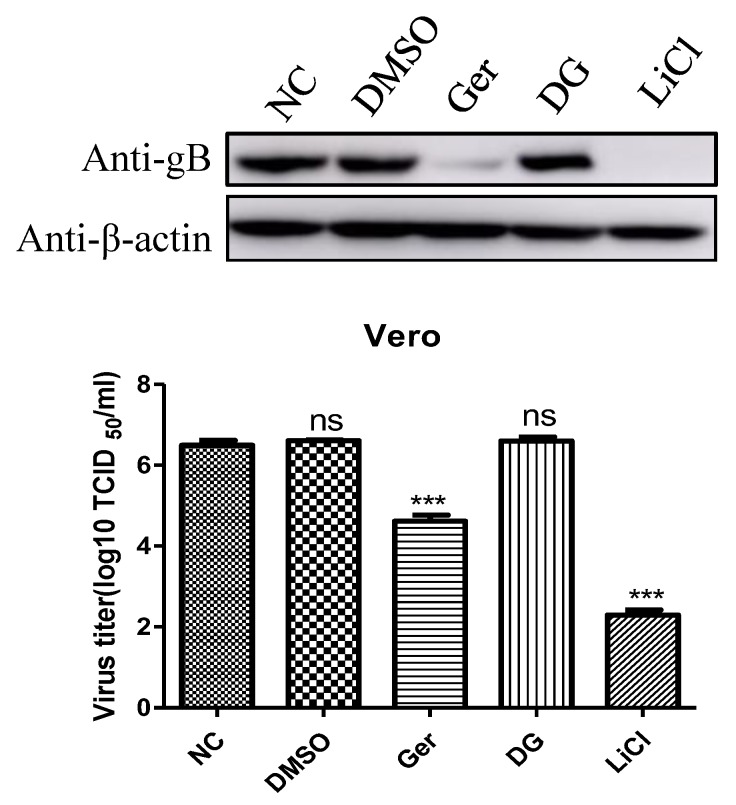
Comparison of antiviral effects of DG, LiCl, and germacrone. Cells were infected with PRV (MOI 0.1) for 1 h, then medium containing different drugs (150 μM of germacrone, 50 mM of LiCl, and 1250 μg/mL of DG) was added. DMSO was used as a control. After 24 h of treatment, the level of gB protein was detected by western blot and the virus yielded in supernatants were determined by TCID_50_ in Vero cells (*** *p* < 0.001).

It has been reported that DG and LiCl inhibit PRV infection of Vero cells [15]. Compared with LiCl, DG did not inhibit PRV infection after virus adsorption, and it seemed that this drug was only viricidal to PRV. Therefore, we explored the antiviral effects of germacone, DG, and LiCl after PRV variant strain infection. As shown in Figure 6, the antiviral effect of germacone is better than that of DG and worse than that of LiCl, measured by western bolt and TCID_50_ at the nontoxic concentration.

**Figure 7:**
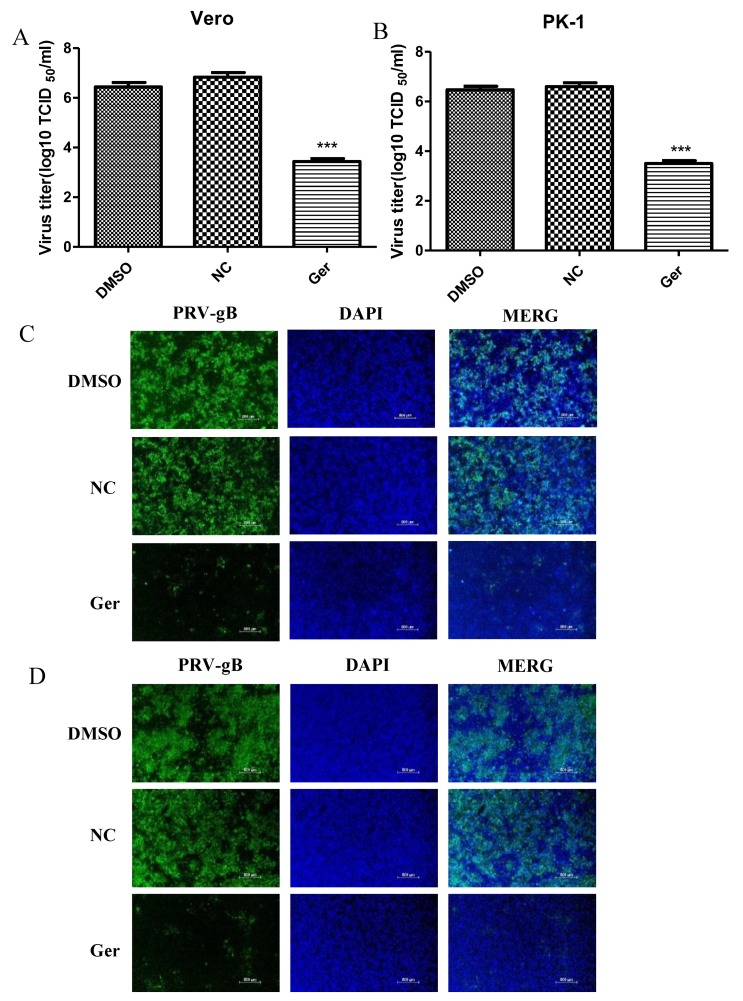
Germacrone reduces the production of PRV vaccine strain Barth K61. (**A**,**B**) Vero (**A**) and PK-1 (**B**) cells were infected with PRV vaccine strain Barth K61 (MOI 0.1) for 1 h. The unbound virus particles were washed with serum-free DMEM, then medium containing 150 μM of germacrone was added. DMSO was used as a control. After 24 h of treatment, virus yielded in supernatants from infected cells was determined by TCID_50_ in Vero cells. (**C**,**D**) The effect of different concentrations of germacrone on PRV, shown by IFA at 24 hpi in Vero cells (**C**) or PK-1 cells (**D**). Scale bar, 250 μm (*** *p* < 0.001).

We also explored the antiviral activity of germacone against PRV vaccine strain Barth K61. Vero cells were infected with PRV vaccine strain Barth K61 at a MOI of 0.1 for 1 h and treated with 150 μM germacrone. The recovered virus yields were determined by TCID_50._ As shown in Figure 7A,B, germacrone treatment significantly suppressed the growth of PRV vaccine strain Barth K61 in Vero or PK-1 cells (Figure 7A,B). In IFA, the fluorescence signals declined after treatment with 150 μM germacrone (Figure 7C,D). This indicates that germacrone treatment inhibits the replication of PRV vaccine strain Barth K61.

## 4. Discussion

Over the past decade, a number of novel viruses emerged in pigs in China and caused huge economic losses [28,29]. As one of them, at the end of 2011, re-emergence PR outbreaks were reported in many pig farms in China. Evidence suggests that the new epidemic is the result of a variant PRV strain [2,30]. The Bartha-K61 vaccine is inadequate to protect pigs from infection with variant PRV [8], and thus antiviral therapeutics may be a critical strategy to control variant PRV damage in the swine husbandry. PRV is an important threat to animals and possibly humans. A case of suspected PRV infection in humans occurred in China in 2017. The patient presented with fever, headaches, and visual impairment [4]. Many countries have also reported PRV interspecies transmission [3]. With the emergence of new viruses that threaten both the pig industry and public health, the discovery of new functions in existing clinical drugs is often used in the screening of modern emergency drugs. Germacrone has an antiviral effect on a variety of viruses, such as IAV, PPV, FCV, and PRRSV [16,20,21,22]. In this study, we investigated the effect of germacrone on PRV variant strain. We found that germacrone could inhibit PRV replication in a dose-dependent manner at the early stage of virus replication by western blot and IFA. We showed that germacrone could not directly kill the virus nor affect the adsorption and invasion of PRV. We also found that it could not affect the expression of nectin-1, nectin-2, and CD155, the receptors of PRV. To further investigate at which phase PRV replication is sensitive to germacrone treatment, cells were exposed to germacrone at various time points. PRV replication was impressed significantly when cells were treated with germacrone at 6 hpi. In addition, we found that the antiviral effect at 12 h was significantly higher than at 6 h, so we concluded that germacrone had the best antiviral effect in the early stage of virus replication, as in the case of PRRSV [16]. We speculate that treatment with germacrone may affect the expression of host genes or cellular factors, some of which may be required for viral replication. Therefore, we speculate that germacrone may inhibit PRV replication by affecting a cell antiviral mechanism.

We explored the anti-PRV virus drugs that have been reported, including a traditional Chinese medicine, DG, and a chemical drug, LiCl. Both DG and germarone belong to traditional Chinese medicine. However, unlike DG which directly kills the virus, germarone plays a role at the early stage of PRV replication and can be used as a therapeutic drug. Although the antiviral effect of germacone is worse than that of LiCl, germacone is safer, considering that resistance develops with the use of chemical drugs like LiCl in clinical applications. We also studied the effect of germarone on the vaccine strain Bartha-K61 and found that it inhibits the replication of PRV vaccine strain Barth K61. So germacrone can be used in vaccinated pigs that are not completely protected against variant type PRV.

Germacrone is a monocyclic sesquiterpenoid compound. Recently, it was found that germacrone has strong antiviral activity, as well as anti-inflammatory and other pharmacological effects potentially useful for clinical applications [31,32]. In particular, the germacrone and oseltamivir combination has a synergistic effect on the inhibition of influenza virus. On the other hand, there are no reports on potential antitumor activity in vivo [33]. The main possible reasons for this phenomenon are: (1) germacrone is mainly extracted and isolated from plants with relatively low yield, therefore the purchase price is high, while the dosage for animal experiments is large; (2) the water solubility of germacrone is relatively poor; and (3) the lack of an antitumor and antivirus mechanism. For all the above reasons, we need to conduct in-depth research on the antiviral mechanism of germacrone, modify the extraction and separation methods to increase the yield, and improve it to make it safer for animal use.

In summary, we report for the first time the inhibitory effect of germacone on the replication of PRV. We show it acts at the early stage of infection and could serve as a potential treatment. However, the antiviral effect of germacone in vivo needs to be further explored.

## Figures and Tables

**Table 1:** Detection and sequencing primers.

Gene	Primer (5′-3′)
*PRV-gB-F*	GTCACCCGCGTGCTGATCGTCT
*PRV-gB-R*	GGCAACCACCGGCGCTACTTT
*Nectin-1-F*	TGGGAAACTCGGCTAAAAGG
*Nectin-1-R*	TGTGGTAGTTGACGATGCAG
*Nectin-2-F*	TACACCTGCGAGTTTGCCACC
*Nectin-2-R*	AAGCGGCTGGTGACGGTGAC
*CD155-F*	TCTGGATTTTGGTGCCCTC
*CD155-R*	TTCTCAAAGCTCTCGTGCTC
*β-actin-F*	CTCCATCATGAAGTGCGACGT
*β-actin-R*	GTGATCTCCTTCTGCATCCTGTC

## Abbreviations

Pseudorabies	PR
Pseudorabies virus	PRV
Negative control	NC
Lithium chloride	LiCl
Diammonium glycyrrhizinate	DG
Inhibits influenza virus	IAV
Porcine parvovirus	PPV
Feline calicivirus	FCV
Porcine reproductive and respiratory syndrome virus	PRRSV
50% effective concentration	EC_50_
50% cytotoxic concentration	CC_50_
50% tissue culture infectious dose	TCID_50_
Indirect immunofluorescence assay	IFA
Multiplicity of infection	MOI
